# Shared decision-making for renal replacement treatment and illness perception in patients with advanced chronic kidney disease

**DOI:** 10.1186/s12911-023-02261-w

**Published:** 2023-08-14

**Authors:** Shih-Ming Hsiao, Mei-Chuan Kuo, Pei-Ni Hsiao, Sin-Hua Moi, Yi-Wen Chiu, Shu-Li Wang, Tzu-Hui Chen, Lan-Fang Kung, Shang-Jyh Hwang, Chia-Lun Lee

**Affiliations:** 1 Department of Nursing, Kaohsiung Medical University Hospital, Kaohsiung Medical University, Kaohsiung, 807 Taiwan; 2 Department of Nephrology, Kaohsiung Medical University Hospital, Kaohsiung Medical University, Kaohsiung, 807 Taiwan; 3 School of Medicine, College of Medicine, Kaohsiung Medical University, Kaohsiung, 807 Taiwan; 4 Graduate Institute of Clinical Medicine, College of Medicine, Kaohsiung Medical University, Kaohsiung, 807 Taiwan

**Keywords:** Shared decision-making, Illness perception, Chronic kidney disease, Renal replacement therapy

## Abstract

**Background:**

Current healthcare trends emphasize the use of shared decision-making (SDM) for renal replacement treatment (RRT) in patients with chronic kidney disease (CKD). This is crucial to understand the relationship between SDM and illness perception of CKD patients. Few studies have focused on SDM and illness perception status of CKD patients and the impact of illness perception on RRT after SDM.

**Methods:**

In this cross-sectional study, we used a questionnaire with purposive sampling from March 2019 to February 2020 at the nephrology outpatient department of a medical center in southern Taiwan. The nephrology medical team in this study used the SHARE five-step model of SDM to communicate with the patients about RRT and Brief Illness Perception Questionnaire (BIPQ) was applied to evaluate illness perception of these patients at the beginning of SDM. According to the SDM decision time, the study participants were classified general and delayed SDM groups. The distribution between SDM groups was estimated using independent two sample t-test, chi-squared test or Fisher’s exact test. The correlation between illness perception and SDM decision time were illustrated and evaluated using Spearman’s correlation test. A p-value less than 0.05 is statistically significant.

**Results:**

A total of 75 patients were enrolled in this study. The average time to make a dialysis decision after initiating SDM was 166.2 ± 178.1 days. 51 patients were classified as general group, and 24 patients were classified as delayed group. The median SDM decision time of delayed group were significantly longer than general group (56 vs. 361 days, *P* < 0.001). Our findings revealed that delayed group was significantly characterized with not created early surgical assess (delayed vs. general: 66.7% vs. 27.5%, *p* = 0.001) compared to general group. The average BIPQ score was 54.0 ± 8.1 in our study. We classified the patients into high and low illness perception group according to the median score of BIPQ. The total score of BIPQ in overall participants might increase by the SDM decision time (*rho* = 0.83, *p* = 0.830) and the linear regression line also showed consistent trends between BIPQ and SDM decision time in correspond cohorts. However, no statistically significant findings were found.

**Conclusions:**

The patients with advanced chronic kidney disease took an average of five and a half months to make a RRT decision after undergoing SDM. Although there is no statistical significance, the trend of illness perception seems correlated with decision-making time. The stronger the illness perception, the longer the decision-making time. Furthermore, shorter decision times may be associated with earlier establishment of surgical access. We need more research exploring the relationship between illness perception and SDM for RRT in CKD patients.

**Supplementary Information:**

The online version contains supplementary material available at 10.1186/s12911-023-02261-w.

## Background

Chronic kidney disease (CKD) is a severe public health problem, imposing a heavy financial burden on healthcare systems worldwide [1]. The ideal process of initiating renal replacement treatment (RRT) should be through joint decision-making of the CKD patients, their family members, and the healthcare team. Shared decision-making (SDM) can help the healthcare team maintain good communication with the CKD patients, help the patients understand the different options of RRT, and then make the most appropriate decision [2, 3]. The American Society of Nephrology recommends that healthcare teams should use SDM to discuss when to start and withdraw hemodialysis with prospective patients [4]. SDM has been shown to improve the quality and consequence of the decision-making process for different diseases, and also to affect the patients’ lifestyles [5]. SDM has also been reported to reduce uncertainty in CKD patients and increase treatment satisfaction [6].

SDM is an approach that integrates the principles of “evidence-based medicine” and “patient-centeredness,“ emphasizing effective communication between clinical professionals and patients by considering the patients’ values, preferences, and expectations [7]. Consequently, patients’ values and preferences can significantly impact medical decisions. Patients’ perception of their illness is influenced by their previous knowledge and experience, including symptoms, etiology, prognosis, and treatment effectiveness. These factors relate to the concept of illness perception, defined as patients’ perception of their illness, which may differ based on their knowledge and experience of the illness [8, 9]. A systematic literature review has shown that illness perception changes over time, with personal control being the most affected by time. However, the findings also indicated that interventions could alter the causal beliefs and personal control of dialysis patients [10].

Current healthcare trends emphasize the use of SDM for RRT in CKD patients. Due to relative slow progression in CKD and dynamic process in SDM, SDM for RRT is not an urgent requirement and has a long consideration period. There is currently no predefined time point for decision-making, and few studies have investigated the timing of the decision to start RRT in CKD patients [11]. While facing disease, patients respond cognitively, emotionally and give personal meaning and ideas to rationalize feelings, which is called illness perception [12]. Illness perception is important, which related to clinical results, an important factor affecting health coping behavior, and may alter the risk factors for CKD patients [8, 13]. Patients with greater illness perception can take effective actions to deal with the disease and then accept and make treatment choices [8].

Accordingly, the aim of this study was to explore the use of SDM for RRT, the illness perception of CKD patients, and the relationships between them. We hope that the results could be used as a reference to promote SDM for RRT in CKD patients.

## Methods

### Study design and sample

In this cross-sectional study, we conducted a questionnaire survey with purposive sampling from March 2019 to February 2020 at the nephrology outpatient department in a medical center in southern Taiwan. The inclusion criteria were: (1) age ≥ 20 years; (2) a diagnosis of CKD stage 5; and (3) having initiated SDM for RRT by a nephrologist. The RRT selection and preimplantation surveyed in December 2020. Patients who did not make an RRT decision prior to the survey were excluded from this study.

**Fig. 1:**
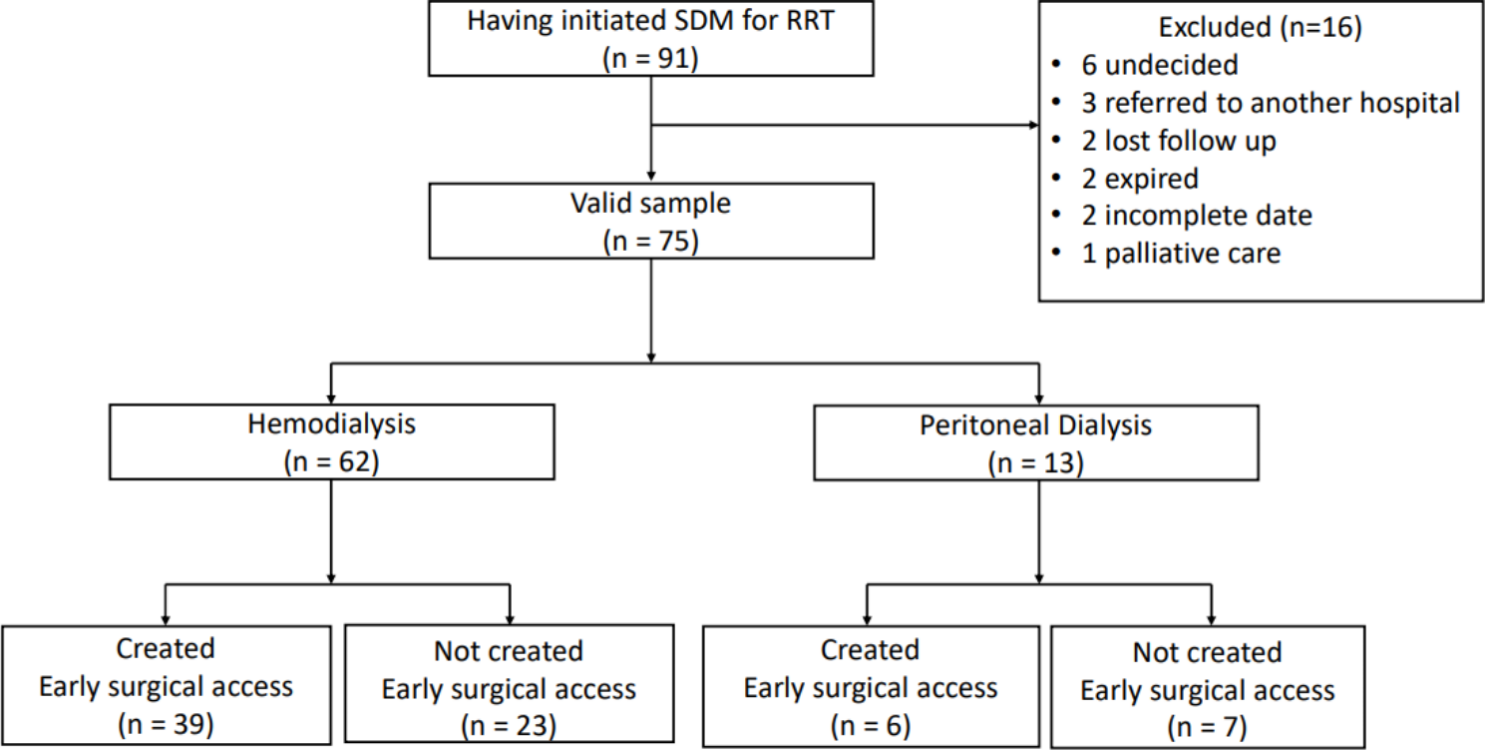
Flowchart of study

According to the factor-analytic methods proposed by A.L. Comrey, estimating sample size based on ten times the number of questions is one of the common strategies in clinical and social psychologists [14]. All nine questions were used in our illness perception questionnaire, thus we enrolled 91 CKD patients at initial to meet the minimum sample size requirements of 90 participants. All patients who initiated SDM for RRT finished the illness perception questionnaire. However, six patients were undecided RRT choice, three patients were referred to another hospital, two patients were lost to follow-up, and two patients were died before December 2020, two patients had incomplete clinical data, and one patient received palliative care. Since our study was designed to investigate the use of SDM in RRT, finally, only 75 (82.4%) patients who made a RRT decision and had received hemodialysis or peritoneal dialysis before the day were analyzed. According to general concept by Statistician, it is acceptable for the attrition rate less than 20%. The flowchart of study and exact patients number involved in each step were illustrated in Fig. 1.

As “created early surgical access” indicated that the patient had established a long-term dialysis vascular access or peritoneal dialysis catheter before the first dialysis session, the item was discriminated as “created” or “not created”.

### Instruments and data collection

The standard process in our outpatient department is to initiate RRT SDM for appropriate CKD patients during a visit to the attending physician while CKD stage 5 and probably had risk of kidney failure within 1 year [15]. Certificated kidney disease educators use the SHARE five-step model of SDM to communicate with the patients about RRT and to follow up and clarify their concerns on an ongoing basis in the outpatient department. Although the time spent by each patient was different. Some patients needed to confirm and clarify in the outpatient department visit, but all the participants have completed the SHARE 5-step model. The SDM SHARE five-step model includes seeking patient participation (Seek), helping patients discover and compare possible treatment options (Help), assessing patient values and preferences (Assess), making decisions with the patient (Reach), and evaluating the patient’s decision (Evaluate) [2]. SDM empowers patients to choose their RRT option, including the dialysis method. After the patient has made their decision, the healthcare provider discuss the possibility of pre-implanting the dialysis circuit to ensure a smooth transition to dialysis. As for timing for dialysis, it is followed by K-DIGO guideline and depends on physician’s judgement [15].

The patients are asked if they would like to fill out the questionnaires when they confirm to perform the SDM. The questionnaires include a characteristics sheet and the Brief Illness Perception Questionnaire (BIPQ) [16] are conducted in a separate room, and medical records are consulted after obtaining the consent of the patients. The educators only explain the questionnaire if the patients state they are unable to understand the content.

The characteristics sheet included demographic variables and laboratory data. The demographic variables included gender, age, marital status, education, employee status, primary caregiver and main medical decision-maker, comorbidity, and the laboratory data included creatinine level and estimated glomerular filtration rate (eGFR) at initial of study. Broadbent et al. reviewed 188 studies using the BIPQ and found that it was suitable for subjects aged 8 to 80. The BIPQ has been translated into 26 languages, is widely used in 36 countries/regions, and for patients with many different diseases, including CKD. The BIPQ has also been shown to have good concurrent validity, predictive validity, differential validity, and test-retest reliability to assess a patient’s feelings about a disease (*r* = 0.42 ~ 0.75) [10], and also good reliability in patients with CKD/end-stage renal disease. The Cronbach α coefficient of this scale for CKD stage 4–5 and post-dialysis patients is 0.93 [17].

### SDM indicators for RRT

Shared decision making for CKD patients is mainly used in the selection of RRT. Through the process of SDM, it helps patients decide the method of dialysis in the future and whether to pre-place the dialysis access before starting dialysis [18].Therefore, the SDM indicators in this study mainly involved three items: (1) the RRT includes dialysis and kidney transplantation. The dialysis methods including hemodialysis and peritoneal dialysis; (2) the decision time, defined as the number of days from when the physician started RRT SDM until the patient made decision for modality of dialysis; and (3) whether surgical dialysis access was created. We collected the above data through medical records. Although kidney transplantation is one choice of the RRT, none of the 75 participants in this study chose kidney transplantation.

Because few previous studies have discussed the ideal number of days for RRT SDM, we ascendingly ranked the patients based on the number of days to make a decision for RRT, then summarizes the differences of RRT decision time between different grouping methods, and finally to select the most appropriate grouping method. Accordingly, we divided the participants into two, three, or four equal groups depend on the SDM decision time for RRT. The grouping details were summarized in Supplementary Table S1. Since the decision time of the top two-thirds patients was closer to the median SDM times, we further classified the top two-thirds participants into general SDM group, and the bottom one-thirds participants were classified as delayed SDM group.

### Illness perception measurements

The BIPQ contains nine questions, which mainly evaluate the patients’ thoughts and emotional reactions to their disease. The eight questions cover eight domains, and each question is rated from 0 to 10 points. The ninth question is an open-ended question in which the patients are asked to list the three most important reasons for their illness, and then the researcher classifies the causes, such as health awareness, disease progression, and lifestyle. The first five of the domains are related to disease, namely disease consequence, timeline (duration of the disease), personal control (self-control of the disease), treatment control (treatment effect), and identity (symptoms caused by the disease). The next two domains are related to emotions, namely concern (degree of concern for the disease), and emotional response (emotions caused by illness, such as anger, fear, or depression), and the eighth domain is understanding (degree of illness comprehensibility). The total score ranges from 0 to 80, with a higher score indicating a stronger feeling for the illness [16]. The average score of the BIPQ in our participants was 54.0 ± 8.1. A previous study suggested that dividing patients into high and low groups according to their illness perception average score was helpful for statistical analysis [19] (Supplementary Table S2).

### Statistical analysis

All statistical analyses were performed using SAS version 9.4 for Windows (SAS Inc., Cary, NC) and R 4.1.2 software (R Core team, 2023, Vienna, Austria). The study variables were summarized using mean (standard deviation), median (interquartile range), or frequency (percentages). The distribution between SDM groups was estimated using independent two sample t-test, chi-squared test, or Fisher’s exact test. The illness perception and total score of BIPQ were summarized using mean and SD, and the difference between SDM groups was estimated using independent two sample *t*-test. The correlation between total score of BIPQ and SDM decision time (days) were illustrated using scatter plot with linear regression line, and the correlation between BIPQ score and SDM decision time were evaluated using Spearman’s correlation test.

### Ethical considerations

This study was reviewed and approved by the Institutional Review Board (Project No. KMUHIRB-E(I)20,180,140) of the study site. According to ethical considerations and privacy protection, before the study, the study participants were fully informed that they could withdraw at any time during the study process and that their right of medical care would not be affected in any way. Data collection was anonymous, and the study results did not reveal the identity of any individual case.

## Results

### Baseline characteristics of the participants

Table 1 summarizes the baseline characteristics of the participants according to the SDM groups. A total of 75 patients were enrolled in this study, including 54 males (72.0%) and 21 females (28.0%), with a mean age of 61.3 ± 12.8 years. As mentioned above, we divided the study participants into general and delayed group based on their number of SDM days for RRT. 51 (68.0%) patients were classified as general group, and 24 (32.0%) patients were classified as delayed group. The SDM decision time for all patients were averaged 166.2 ± 178.1 days, with a median of 84 days and interquartile range between 32 and 280 days. Unsurprisingly, the median SDM decision time of delayed group were significantly longer than general group (56 vs. 361 days, *p* < 0.001). Although delayed group has elder mean age and higher proportion in elderly group (age ≥ 65 years), no statistically significant were found between general and delayed group. However, female patients showed a significant higher proportion in delayed group compared to general group (45.8% vs. 19.6%, *p* = 0.018). Apart from this, both groups showed similar distribution of the summarized characteristics including education, marital status, employee status, primary caregiver, and main decision maker. In comorbidity investigation, 30 (40.0%), 54 (72.0%), 15 (20.0%), and 7 (9.3%) patients were characterized by diabetes mellitus, hypertension, cardiovascular disease, and stroke, respectively. A significant higher proportion of cardiovascular disease were found in general group compared to delayed group (27.5% vs. 4.2%, *p* = 0.028), while similar proportion were found in diabetes mellitus, hypertension, and stroke between both groups. For laboratory measurements, the mean creatinine level at initial of all patients was 7.1 ± 2.1 mg/dl, and the eGFR was 7.9 ± 2.2 mL/min/1.73 m^2^. Delayed group showed significant lower initial creatinine level compared to general group (7.5 ± 2.2 vs. 6.1 ± 1.5 mL/min/1.73 m^2^, *p* = 0.003).

**Table 1:** Baseline characteristics of participants according to the SDM groups (n = 75)

Characteristics	Overall, n = 75	General group, n = 51	Delayed group, n = 24	Statistics	*p*
SDM for RRT (days)					
Mean ± SD	166.2 ± 178.1	59.9 ± 47.7	391.9 ± 137.5	-11.51	**< 0.001**
Median (interquartile range)	84 (32–280)	56 (21–88)	361 (302–443)		
Age (years)	61.3 ± 12.8	60.3 ± 12.5	63.5 ± 13.4	-0.98	0.334
Age group				3.00	0.086
< 65	45 (60.0%)	34 (66.7%)	11 (45.8%)		
≥ 65	30 (40.0%)	17 (33.3%)	13 (54.2%)		
Gender				5.60	**0.018**
Male	54 (72.0%)	41 (80.4%)	13 (54.2%)		
Female	21 (28.0%)	10 (19.6%)	11 (45.8%)		
Education (years)				0.78	0.378
≥ 12 (Undergraduate/Graduate)	32 (42.7%)	20 (39.2%)	12 (50.0%)		
< 12 (High school or below)	43 (57.3%)	31 (60.8%)	12 (50.0%)		
Marital status				0.02	0.877
Married	54 (72.0%)	37 (72.5%)	17 (70.8%)		
Single/Widowed	21 (28.0%)	14 (27.5%)	7 (29.2%)		
Employee status				0.01	0.921
Employed	35 (46.7%)	24 (47.1%)	11 (45.8%)		
Unemployed	40 (53.3%)	27 (52.9%)	13 (54.2%)		
Primary caregiver				< 0.01	1.000
Own	66 (88.0%)	45 (88.2%)	21 (87.5%)		
Others	9 (12.0%)	6 (11.8%)	3 (12.5%)		
Main decision maker				< 0.01	0.705
Own	67 (89.3%)	46 (90.2%)	21 (87.5%)		
Others	8 (10.7%)	5 (9.8%)	3 (12.5%)		
Comorbidity					
Diabetes mellitus	30 (40.0%)	23 (45.1%)	7 (29.2%)	1.70	0.189
Hypertension	54 (72.0%)	38 (74.5%)	16 (66.7%)	0.50	0.480
Cardiovascular disease	15 (20.0%)	14 (27.5%)	1 (4.2%)	< 0.01	**0.028**
Stroke	7 (9.3%)	6 (11.8%)	1 (4.2%)	< 0.01	0.419
Laboratory measurements					
Initial creatinine	7.1 ± 2.1	7.5 ± 2.2	6.1 ± 1.5	3.10	**0.003**
Initial eGFR	7.9 ± 2.2	7.6 ± 2.3	8.5 ± 1.9	-1.80	0.084

Statistics value and *p*-value are estimated using independent two sample t-test, chi-squared test or Fisher’s exact test

### Illness perception of study participants

The illness perception measured by BIPQ is presents in Table 2. The average BIPQ score of all patients was 54.0 ± 8.1, and the top ranked scores for a single domain was duration of disease (timeline: 8.6 ± 2.4), followed by degree of concern for the disease (concern: 8.4 ± 1.9), and treatment effect (treatment control: 7.0 ± 1.8). Although delayed group showed overall lower BIPQ score compared to general group, no significant findings were found between groups. Overall, delayed group showed lower perception to disease consequence, timeline, personal control, identity, and emotional response compared to general group, but higher perception to treatment control, concern, and understanding. Consistently, no significant difference was found in each domain of BIPQ between both groups.

**Table 2:** Illness perception of study participants according to the SDM groups (n = 75)

Terms	Overall, n = 75	General group, n = 51	Delayed group, n = 24	*t*	*p*
BIPQ total score	54.0 ± 8.1	54.2 ± 8.2	53.6 ± 8.2	0.27	0.787
Related to disease					
Timeline	8.6 ± 2.4	8.9 ± 1.8	8.1 ± 3.3	1.10	0.269
Treatment control	7.0 ± 1.8	6.8 ± 1.7	7.3 ± 1.9	-1.20	0.249
Personal control	6.2 ± 1.8	6.2 ± 1.7	6.1 ± 2.0	0.24	0.812
Identity	5.3 ± 2.4	5.4 ± 2.5	5.2 ± 2.4	0.21	0.838
Disease consequence	5.1 ± 2.5	5.3 ± 2.6	4.9 ± 2.2	0.68	0.497
Related to emotions					
Concern	8.4 ± 1.9	8.2 ± 1.9	8.8 ± 1.8	-1.30	0.203
Emotional response	6.4 ± 2.6	6.6 ± 2.5	6.1 ± 2.9	0.67	0.505
Understanding	6.9 ± 2.0	6.8 ± 1.9	7.0 ± 2.3	-0.41	0.684

All *t*-value and *p*-value are estimated using independent two sample t-test

In response to the ninth open-ended question of BIPQ, 87.6% of participants provided answers. The research team analyzed and ranked the most frequently cited causes of their disease. These causes included lack of health awareness (neglect of physical care, irregular examination/medication, and lack of health knowledge) which was reported by 25 participants (32.1%), chronic disease progression (including diabetes, high blood pressure, and hyperlipidemia) which was reported by 23 participants (29.5%), and unhealthy lifestyle (unhealthy eating habits, smoking, lack of exercise, and poor treatment compliance) which was reported by 20 participants (25.6%).

### Differences in illness perception, dialysis method, and surgical access creation between the SDM groups

Table 3 presents the distribution of illness perception, dialysis method, and surgical access creation between SDM groups. We classified the patients into high perception (BIPQ ≥ 54) and low perception (BIPQ < 54) group according to the median score of BIPQ (see Table 2). As expected, delayed group showed higher proportion in low perception to illness, but not significant difference was found compared to general group. Although delayed group showed higher proportion to receive peritoneal dialysis, no statistically significant difference was found compared to hemodialysis group. Nonetheless, our findings noted that delayed group was significantly characterized with not created early surgical assess (delayed vs. general: 66.7% vs. 27.5%, *p* = 0.001) compared to general group.

**Table 3:** Illness perception, dialysis method and early surgical access of study participants according to the SDM groups (n = 75)

Characteristics	Overall, n = 75	General group, n = 51	Delayed group, n = 24	χ^2^	*p*
Illness perception				0.33	0.566
High perception (BIPQ ≥ 54)	38 (50.7%)	27 (52.9%)	11 (45.8%)		
Low perception (BIPQ < 54)	37 (49.3%)	24 (47.1%)	13 (54.2%)		
Dialysis methods				< 0.01	0.100
Hemodialysis (HD)	62 (82.7%)	45 (88.2%)	17 (70.8%)		
Peritoneal dialysis (PD)	13 (17.3%)	6 (11.8%)	7 (29.2%)		
Early surgical access				10.00	**0.001**
Not created	30 (40.0%)	14 (27.5%)	16 (66.7%)		
Created	45 (60.0%)	37 (72.5%)	8 (33.3%)		

χ^2^ and *p*-value are estimated using chi-squared test. BIPQ, Brief Illness Perception Questionnaire

### Correlation between illness perception and SDM decision time

**Fig. 2:**
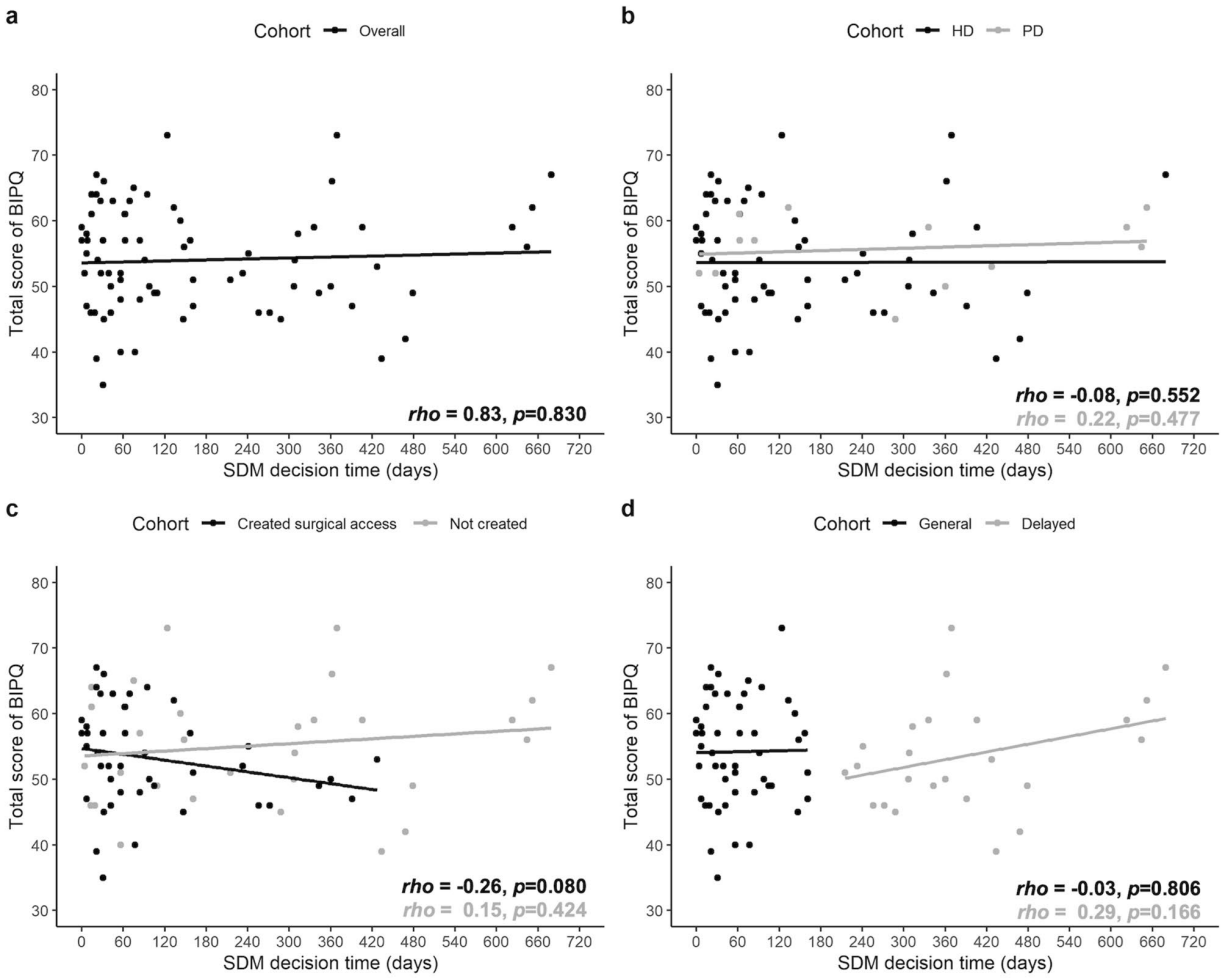
Correlation between illness perception and SDM decision time in (a) overall cohort, and different (b) dialysis methods, (c) surgical access creation, and (d) SDM groups

Figure 2 summarizes the correlation between illness perception score and SDM decision time in days. Overall, the total score of BIPQ in overall participants might increase by the SDM decision time (*rho* = 0.83, *p* = 0.830) as shown in Fig. 2a. However, after splitting the patients according to the dialysis methods, early surgical access creation or not, and general and delayed SDM groups, our results indicate that the BIPQ score of patients characterized with choosing hemodialysis (Fig. 2b, *rho* = -0.08, *p* = 0.552), early created surgical access (Fig. 2c, *rho* = -0.26, *p* = 0.080), and general SDM group (Fig. 2d, *rho* = -0.03, *p* = 0.806) were negatively correlated with SDM decision times. While the patients who characterized with choosing peritoneal dialysis, not created surgical access, and delayed SDM group showed positive correlation between BIPQ score and SDM decision times. In addition, the linear regression line also showed consistent trends between BIPQ and SDM decision on time in correspond cohorts as Fig. 2a. However, no statistically significant findings were found.

In conclusion, the correlation between BIPQ score and SDM decision time were more in line with expectation in general SDM group, but no similar findings were found in delayed SDM group. Furthermore, an early surgical access creation might also potentially correlate to shorten the SDM decision time in study participants.

## Discussion

This study is to investigate the relationship between illness perception and the use of SDM for RRT in CKD patients. The research team hypothesized that there is a significant association between them. Although the obtained results did not reach statistical significance, a discernible trend was observed. More details were discussed in the following.

### SDM consideration time of the CKD patients

The SDM decision time in this study for all patients were averaged 166 days. One paper mentioned after the initiation of SDM by the healthcare team, patients took an average of 76.8 days (SD = 87.1) from the initiation of SDM to confirmation of dialysis and establishment of dialysis vascular access or peritoneal dialysis catheter [4]. Compared with usual care and traditional education, SDM has been shown to increase patients’ ability to take an active role in their health and medical care and to make individualized and informed treatment decisions based on their values and beliefs [2]. However, introducing the SDM when educating patients about RRT cannot shorten the decision time of modality of RRT, as finding in this study. Moreover, some patients in the delayed decision group took more than 1 year to decide on dialysis.

Another important finding in this study revealed that female patients showed a higher proportion in delayed group compared to general group in this study. Many factors can affect medical decision-making in CKD patients. The literature mentions that women with poor health-related quality of life, and low socioeconomic status as barriers to medical decision-making [20]. This may partially explain the reason for higher proportion of female in delayed group.

Our study also found a significant higher proportion of cardiovascular disease patients in general group compared to delayed group (27.5% vs. 4.2%, *p* = 0.028) and delayed group showed significant lower initial creatinine level compared to general group (7.5 ± 2.2 vs. 6.1 ± 1.5 mL/min/1.73 m2, *p* = 0.003). These findings are compatible with current clinical practices. Specifically, patients diagnosed with cardiovascular disease are more likely to be vigilant about their health and tend to make prompt medical decisions, resulting in reduced decision-making time. Conversely, patients with low initial creatinine levels may assume that they have more time to contemplate their options, which could potentially result in delayed decision-making.

### Illness perceptions of the CKD patients

The average BIPQ score in our study was 54.0 ± 8.1, and three highest-scoring items were timeline (8.6 ± 2.4), concern (8.4 ± 1.9), and treatment control (7.0 ± 1.8). Jansen et al. (2010) conducted an illness perception survey of 109 adults with CKD stages 4 ~ 5, and their reported scores for timeline (9.3 ± 1.7) and treatment control (6.8 ± 2.9) are similar to our results [21]. The timeline score in Meuleman et al.’s study was 8.2 ± 1.4, which is also similar to our result [22]. In general, kidney disease is a lifelong incurable disease, and all patients with CKD stages 4 to 5 express concern about the duration of the disease. The patients with positive awareness of kidney disease have been shown to have better socio-psychological and clinical outcomes, including higher quality of life, treatment compliance, and survival rate [23].

At present, few studies have investigated the open question of illness perception. Patients’ views on the causes of illness are usually based on personal experience, discussion with and opinions from significant others [24]. The patients in this study believed that the main causes of illness were insufficient health awareness, chronic disease progression, and poor lifestyle. Personal control has been reported to be the most changeable item over time among all illness perception items [10]. If the healthcare team could provide effective strategies to change the patient’s illness perception and lifestyle, the patient’s health awareness might be enhanced, and the attitude towards the disease and coping strategies could be changed. This could then help CKD patients to mentally accept their situation and adjust their lifestyle after a diagnosis of kidney disease [25, 26]. Hofstede et al. and Koizumi et al. reported that illness perception, the provision of care information by the healthcare team, and significant interactions in healthcare relationships were the most important factors affecting the implementation of SDM in different kinds of diseases or healthcare systems. Before performing SDM, healthcare providers should understand whether the patients and their families are experiencing negative emotions, as this would affect their ability to make appropriate medical decisions [25, 26].

### Differences in illness perception, dialysis method, and surgical access creation between the SDM groups

The BIPQ scores of patients undergoing hemodialysis (rho = -0.08, *p* = 0.552), those who did not receive an early surgical access (rho = -0.26, *p* = 0.080), and those in the general SDM group (rho = -0.03, *p* = 0.806) were inversely associated with their decision-making time for SDM. Furthermore, our results showed that patients in the delayed group were significantly more likely to lack an early surgical access (66.7% vs. 27.5% in the general group, *p* = 0.001). In contrast, patients characterized by peritoneal dialysis, the absence of a created surgical access, and the delayed SDM group demonstrated a positive correlation between BIPQ score and decision-making time for SDM.

A previous study reported that PD patients had stronger illness perception and perceived their illness to be more manageable compared to HD patients [27]. This may be attributed to PD patients being less reliant on healthcare professionals after treatment. Owing to these characters and influence by culture, the proportion of new peritoneal dialysis patients in Taiwan in 2021 is low (9.4%) [28]. In our study, although only 13 cases chose peritoneal dialysis, which still higher than proportion of new peritoneal dialysis patients in Taiwan (17.3% vs. 9.4%). This finding suggests that SDM might enhance CKD patient chose PD instead of HD. Furthermore, a lower proportion of patients underwent peritoneal dialysis prior to surgical access implantation. Further investigations with larger sample sizes and longer durations are required to validate these results.

### Correlation between illness perception and SDM decision time

In this study, the delayed group had lower BIPQ scores compared to the general group. The overall BIPQ total score for all participants showed a positive correlation with the increase in SDM decision time (rho = 0.83, *p* = 0.830), and the linear regression analysis demonstrated a consistent trend-response association between BIPQ and SDM decision times. However, none of these findings were statistically significant. Although no significant association was found between illness perception and decision-making time in patients with CKD in our study, a discernible trend was observed, which necessitates further investigation.

Medical decision-making is a dynamic process that evolves over time. SDM is a patient-centered care communication strategy between healthcare providers and patients which involves patients’ autonomy and empowerment [7]. CKD patients should be empowered to achieve health outcomes and life goals those are meaningful and important to them [29]. Patients with CKD constantly weigh the pros and cons of different medical decisions, and healthcare providers need to observe and comprehend the patient’s disease perception during this process. Relevant information should be provided to improve the patient’s decision-making ability [30]. Previous studies have shown that SDM improves decision-making quality, knowledge, and illness perception in patients with diabetes. SDM is more effective in patients with strong illness perception [31, 32].

According to a systematic review, the most frequent barriers to SDM implementation are time constraints, perceived unsuitability of SDM due to patient characteristics, and the clinical context [32]. The illness perception of patients may vary and change with clinical status and treatment stage. Patients with dialysis access have been found to spend significant amounts of time discussing and considering the issue with their physicians before accepting dialysis [17, 33]. The effectiveness of communication between patients, families, and medical teams within a limited time depends on the experience of the clinical decision-making team. Even if there is no significant correlation between illness perception and SDM, strategies to improve communication with medical teams can still be explored to enhance the benefits of SDM.

Patients’ beliefs, understanding, and emotional responses to their illness significantly influence their behavior and decision-making. Recognizing and addressing illness perceptions can further contribute to improve treatment adherence and timely activation of RRT when needed [7–9]. Thus, our study expected assessing patient perceptions and preferences using BIPQ tool rather than solely relying on decision outcomes and procedural aspects. Certainly, the key principles of SDM, such as patient autonomy and empowerment would be necessary to further explore the relationship between disease perception and SDM in CKD patients [29]. We have discussed this issue as one of our study limitations and further research direction.

This study still has several limitations that should be noted. First, the timing of SDM initiation in this study was at the discretion of the attending physician, and different physicians may have different considerations, which may have influenced the results. Additionally, the progression of patients’ conditions is variable and may also have affected the findings. Moreover, this was a purposive sampling study conducted at a single medical center in southern Taiwan, which may limit the generalizability of the results. Therefore, further research involving multiple study sites and a larger patient population is necessary to enhance the validity of our findings. We also recommend that future studies employ prospective, longitudinal design, and explore different time points, treatment trajectories, and illness perception measurements both before and after SDM intervention in patients with CKD stage 5.

## Conclusion

This study aimed to investigate the status of SDM, illness perception, and their relationship in patients with CKD. The results showed that the patients took approximately 2 months to more than a year to decide on the dialysis method. We also found a possible positive correlation between SDM decision time and BIPQ total score in the overall participants, and consistent trends were observed between BIPQ and SDM decision time in corresponding cohorts. Although no statistical significance was found, a trend was observed between illness perception and decision-making time, indicating that stronger illness perception was associated with longer decision-making time. In addition, our study participants who made decisions faster tended to have earlier establishment of surgical access. Further research is needed to explore the relationship between illness perception and shared decision-making in CKD patients.

### Electronic supplementary material

Below is the link to the electronic supplementary material.

Supplementary Material 1

Supplementary Material 2

## Data Availability

The datasets generated and/or analysed during the current study are not publicly available due to information shared by the medical team but are available from the corresponding author on reasonable request.

## List of abbreviations

BIPQ: Brief Illness Perception Questionnaire
CKD: Chronic kidney disease
RRT: Renal replacement treatment
SDM: Shared decision-making
