# Supplementary material for: Shared decision-making for renal replacement treatment and illness perception in patients with advanced chronic kidney disease
Source: BMC Med Inform Decis Mak. 2023 Aug 14;23:159. doi: 10.1186/s12911-023-02261-w (PMC10426182; doi:10.1186/s12911-023-02261-w)
Supplement: Supplementary file 1 — Supplementary Material 1 [file 12911_2023_2261_MOESM1_ESM.docx]

**Supplementary Table S1. Different grouping methods of the number of days for RRT SDM.**

| **No. of groups** | **n** | **mean** | **±SD** | **Cutoff point category** |
| --- | --- | --- | --- | --- |
| 2 Groups | 38 | 36.7 | 24.7 | 0~3M |
|  | 37 | 299.4 | 169.0 | >3M |
| 3 Groups | 25 | 21.4 | 13.3 | 0~1.5M |
|  | 26 | 97.3 | 37.5 | >1.5M~5M |
|  | 24 | 391.9 | 137.5 | >5M |
| 4 Groups | 19 | 16.0 | 9.9 | 0~1M |
|  | 19 | 57.3 | 16.0 | >1M~3M |
|  | 19 | 167.2 | 63.8 | >3M~9M |
|  | 18 | 438.9 | 126.5 | >9M |

RRT, renal replacement treatment; SDM, shared decision-making; M, month
