# Supplementary material for: Shared decision-making for renal replacement treatment and illness perception in patients with advanced chronic kidney disease
Source: BMC Med Inform Decis Mak. 2023 Aug 14;23:159. doi: 10.1186/s12911-023-02261-w (PMC10426182; doi:10.1186/s12911-023-02261-w)
Supplement: Supplementary file 2 — Supplementary Material 2 [file 12911_2023_2261_MOESM2_ESM.docx]

**Supplementary Table S2. The high and low BIPQ score groups.**

| **BIPQ Grouping** | **n** | **mean BIPQ score** | **±SD** |
| --- | --- | --- | --- |
| Low score group  (Score <54) | 37 | 47.2 | 4.3 |
| High score group  (Score ≥54) | 38 | 60.6 | 4.8 |

BIPQ, Brief Illness Perception Questionnaire
